# Misuse of Anticholinergic Medications: A Systematic Review

**DOI:** 10.3390/biomedicines10020355

**Published:** 2022-02-01

**Authors:** Stefania Chiappini, Alessio Mosca, Andrea Miuli, Francesco Maria Semeraro, Gianluca Mancusi, Maria Chiara Santovito, Francesco Di Carlo, Mauro Pettorruso, Amira Guirguis, John Martin Corkery, Giovanni Martinotti, Fabrizio Schifano, Massimo Di Giannantonio

**Affiliations:** 1 Department of Neurosciences, Imaging and Clinical Sciences, Università degli Studi G. D’Annunzio, 66100 Chieti, Italy; alessio.mosca909@gmail.com (A.M.); andreamiuli@live.it (A.M.); francescosemeraro911@gmail.com (F.M.S.); g.mancusi@hotmail.com (G.M.); mariachiarasantovito@ymail.com (M.C.S.); francesco.dic@hotmail.it (F.D.C.); mauro.pettorruso@hotmail.it (M.P.); giovanni.martinotti@gmail.com (G.M.); digiannantonio@unich.it (M.D.G.); 2 Psychopharmacology, Drug Misuse and Novel Psychoactive Substances Research Unit, School of Life and Medical Sciences, University of Hertfordshire, Hatfield AL10 9AB, UK; j.corkery@herts.ac.uk (J.M.C.); f.schifano@herts.ac.uk (F.S.); 3 Medical School, The Grove, Swansea University, Swansea SA2 8PP, UK; amira.guirguis@swansea.ac.uk

**Keywords:** anticholinergic drugs, drug misuse, drug abuse, drug diversion

## Abstract

(1) Background: Over the last decade, misuse and diversion of medications has appeared to be increasingly concerning phenomena, including a range of different molecules. As current knowledge on the abuse of centrally acting anticholinergics is limited, the aim of the present study is to review the relevant published data, focusing on the following molecules: benztropine, biperiden, scopolamine, orphenadrine, and benzhexol/trihexyphenidyl (THP). (2) Methods: A systematic literature review was carried out using Pubmed, Scopus, and Web of Science databases following the Preferred Reporting Items for Systematic Reviews and Meta-Analyses (PRISMA). Research methods were registered on PROSPERO (CRD42021257293). (3) Results: A total of 48 articles, including case reports, surveys, and retrospective case series analyses, were included. Most articles focused on benzhexol/THP (*n* = 25), and benztropine (*n* = 4). The routes of administration were mostly oral, and macrodoses together concomitant illicit drugs, e.g., cocaine, have been recorded. Toxidromes included both physical (e.g., tachycardia, tachypnoea, dilatated pupils, dry skin, urinary retention, ataxia, etc.) and psychiatric symptoms (e.g., anxiety, agitation, delirium, etc.). Fatal outcomes were very rare but reported. (4) Conclusion: Results from the present study show that anticholinergic misusing issues are both widespread worldwide and popular. Considering the potential adverse effects associated, healthcare professionals should be vigilant and monitor eventual misusing issues.

## 1. Introduction

### 1.1. Abiuse of Medications

The use of medications for purposes other than medical, such as recreational or enhancement purposes, refers to an increasingly reported phenomenon, known as “pharming”, defining the non-medical use of prescription (e.g., pain relievers, tranquilizers, stimulants, sedatives, etc.) and over-the-counter (OTC) drugs (e.g., loperamide, promethazine, antitussive cough syrups, etc.), either on their own or in combination with other licit or illicit substances [1] and outside of accepted medical guidelines [2]. In the past decades, among prescription drugs recorded, several anticholinergic drugs, known anecdotally to be misused or already reported through literature by online drug user websites and fora, have emerged as abused and diverted [1,2,3].

### 1.2. Abuse of Anticholinergic Medications

The widespread use of anticholinergic agents has been mostly related to their use to alleviate extrapyramidal symptoms in patients receiving neuroleptics for psychosis since the 1960s. However, although the new generation of atypical neuroleptics available is relatively safe on this point of view, anticholinergics are still widely prescribed. Data regarding the prevalence of anticholinergic abuse in the general population are poor, and most prevalence studies refer to mentally ill subjects. Regarding the abuse of anticholinergics in the psychiatric population, it varies widely, going from levels of abuse as high as 34% [4] to only 6.5% [5]. Moreover, data might suffer from the possibility of underdiagnosis, as anticholinergic intoxication might often be mistaken for manifestations of primary psychiatric disorders or other organic diseases [3]. Data drawn from the Norwegian Prescription Database recorded the main consumers of anticholinergic antiparkinsonian drugs were patients using antipsychotic medication, outnumbering patients suffering from Parkinson’s disease by more than 20 to 1. In this study, although the abuse of benzodiazepine tranquilizers was also recorded among patients using antipsychotics, there were no clear indications of abuse of anticholinergics, even among patients who were strongly suspected of abusing benzodiazepines [6]. A case series collecting a number of 40 abusers of anticholinergic drugs attending Oxford hospitals between 1980 and 1982 reported that 28 of them were psychiatric patients on treatment with neuroleptics [7]. Similarly, an American editorial alerted on the abuse of anticholinergic agents, routinely used in psychiatry to treat the extrapyramidal side effects of antipsychotic medications in Jacksonville, Florida, causing an increasing number of evaluations of subjects with chronic mental illnesses in the Emergency Departments on a daily basis [8]. Despite the above-mentioned studies, poor information is available on the abuse of anticholinergic agents, and in most cases, they are partial or limited to case reports/series.

Aims of the study: The current review aimed at: (i) systematically studying the current literature on the misuse of some anticholinergic drugs, including the following molecules: scopolamine, benztropine, biperiden, orphenadrine, and benzhexol/trihexyphenidyl (THP); (ii) describing patterns of anticholinergics’ misuse and eventual related toxicity symptoms; and (iii) better understanding the psychotropic molecular mechanisms underlying their recreational use.

## 2. Materials and Methods

### 2.1. Systematic Review Procedures

A systematic electronic search was performed on 29 November 2021 on PubMed, Scopus, and Web of Science (WoS) databases. The following search strategies have been used, respectively in PubMed and WoS (“anticholinergic” OR “antimuscarinic” OR “scopolamine” OR “benztropine” OR “biperiden” OR “orphenadrine” OR “benzhexol” OR “trihexyphenidyl”) AND (“abuse” OR “misuse” OR “diversion”) NOT animals NOT review; in Scopus: (TITLE-ABS-KEY (“anticholinergic”) OR TITLE-ABS-KEY (“antimuscarinic”) OR TITLE-ABS-KEY (“scopolamine”) OR TITLE-ABS-KEY (“benztropine”) OR TITLE-ABS-KEY (“biperiden”) OR TITLE-ABS-KEY (“orphenadrine”) OR TITLE-ABS-KEY (“benzhexol”) OR TITLE-ABS-KEY (“trihexyphenidyl”) AND TITLE-ABS-KEY (“abuse”) OR TITLE-ABS-KEY (“misuse”) OR TITLE-ABS-KEY (“diversion”) AND NOT TITLE-ABS-KEY (animals) AND NOT TITLE-ABS-KEY (review)). Anticholinergics were selected here on the basis of previously available data on their abuse and diversion, as recorded anecdotally to be misused or already reported through literature by online drug user websites and fora.

The systematic review was structured in accordance with the PRISMA [9,10] and PROSPERO [11] guidelines. Identified studies were assessed at the title/abstract and full-text screening against eligibility criteria.

### 2.2. Data Synthesis Strategy

**Figure 1:**
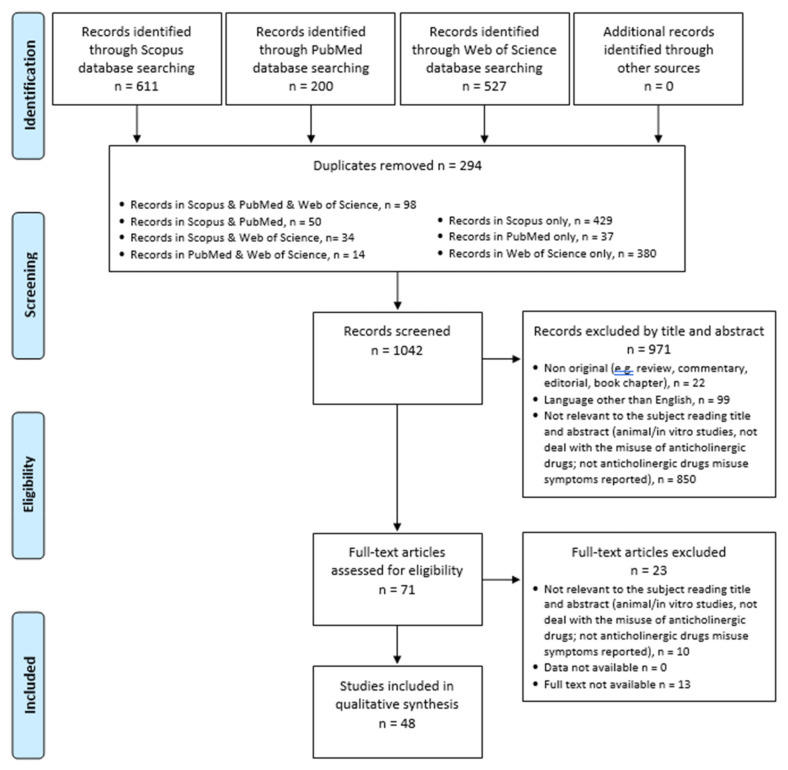
PRISMA flow diagram.

Data were extracted by *n* = 3 investigators (AM, AM, GM/ Gianluca Mancusi, and MCS) supervised by SC and MP; doubtful cases were discussed by the professors GM (Giovanni Martinotti), MdG, and FS. The exclusion criteria were the following: (1) non-original research (e.g., review, commentary, editorial, and book chapter); (2) non-full-text articles (e.g., meeting abstract); (3) language other than English; (4) animal/in vitro studies; (5) articles not dealing with the misuse of anticholinergic drugs; (6) articles without anticholinergic drugs misuse symptoms reported. Removing duplicate articles (*n* = 294) from a total of 1338 papers (PubMed = 200; Scopus = 611; WoS = 527), 1042 records have been screened, and among these, some 850 were not relevant to the subject as they were not dealing with the misuse of anticholinergic drugs, including articles focusing on the misuse of antihistamine drugs with anticholinergic effects and the misuse of datura alkaloids, articles without anticholinergic drug misuse symptoms reported, a number of 99 were not written in English, and 22 were non-original articles (e.g., review, metanalysis, commentary, letter to the editor without data available, and book chapter). Of the 71 full-text articles assessed for eligibility, 23 did not match the inclusion criteria for our review. Finally, 48 articles were included (Figure 1). All these research methods were approved by PROSPERO (identification code CRD42021257293).

## 3. Results

### 3.1. Benzhexol/Trihexyphenidyl (THP)

Benzhexol/Trihexyphenidyl (THP) was the most recorded abused anticholinergic drug (Table 1). It has been covered by twenty-five articles, of which twelve were case reports [12,13,14,15,16,17,18,19,20,21,22,23], six case series [24,25,26,27,28,29], three observational study [30,31,32], two case control [5,33], one cohort study [34], and one survey [35]. Among the 12 subjects reported in the case reports, only two were female [21,22], while the overall age ranged from 19 [13] to 59 [21]. Similarly, case series reported on adult males, ranging from 10.6 [27] to 35 [25] years. Remanent studies also recorded cases of male abusers (Table 1). The route of administration was always oral, but macro dosages have been recorded [28] (Table 1). With regard to the psychiatric comorbidity, schizophrenia emerged as the most recorded [17,20,24,25,26,28,31,32], followed by depression [12,13,14,23,31], substance use disorder [24,25,31,33], schizoaffective disorder [18,28,32], delusional disorder/psychotic disorder [21,22,29], antisocial personality disorder and/or conduct disorder [5,27,31], borderline personality disorder [24,31], adjustment disorder [15,31], and obsessive compulsive disorder [12,30]. Bipolar disorder [31], mixed personality disorder [24], anxiety [13], factitious disorder [32], schizoid disorder [33], unspecific mood disorder [32], attention deficit hyperactivity disorder (ADHD) [27], learning and intellectual disability [27], and intermittent explosive disorder [30] were reported by only one study (Table 1). Finally, three studies reported unspecified mental illness [27,33,34]. Regarding the recorded psychiatric effects, disturbances of perception, in particular hallucinations/illusions, were the most frequently reported [14,15,17,18,19,20,24,27,28,31,35] (Table 1). Eleven articles reported irritability/aggressiveness/nervousness and/or psychomotor agitation [12,15,16,22,25,27,28,29,32,34,35], and nine articles euphoria [12,13,17,18,20,24,28,34,35]. Psychosis/thought disorder was described in seven articles [5,14,19,20,24,31,34], and six articles reported a sedative/relaxing effect [17,23,24,29,30,34]. Disorientation/attention problems, confusion, and concentration/memory disorders were also reported by six articles [15,16,23,25,31,35]. Anxiety and symptoms related to mood alterations [5,15,20,22,24,35] have been described. Medical comorbidity was not recorded in most cases, but extrapyramidal side-effects of neuroleptics [25], cerebral palsy [15], weight loss [12], essential tremor [16], headache, and recurrent abdominal pain [29] were reported. Regarding physical symptoms asscoiated to the abuse of the drug, tachycardia [12,16,17,29,31,35], visual symptoms [12,13,15,25,31], dry mouth [15,20,23,35], headache [16,25,29], movement disorders (including dyskinesia, extrapyramidal symptoms, ataxia) [17,25,32,33], and gastrointestinal symptoms [20,23,35] were the most recorded ones. Licit and illicit substances were associated to the abuse of benzhexol/THP, including, in order, benzodiazepines [18,24,25,28,29,31,33,34], alcohol [5,17,25,30,31,34,35], cannabis [5,17,28,30,31,34], amphetamines [17,28,31,33], heroin/opiates [24,30,31], hallucinogens, e.g., dietylamide lisergic acid (LSD) and phencyclidine (PCP) [17,28,31], nicotine [29,34,35], and cocaine [30,31]. As for the outcome recorded, it was very heterogeneous; it is worth mentioning the strategy of scaling down/interrupting benzhexol/THP [19,23] with the appearance of withdrawal syndrome [16,18,26] treated using benzodiazepines [15,19], neuroleptics [17], or a combination of the two [22,28] (Table 1).

### 3.2. Benztropine

The second most abused molecule was benztropine, which was recorded in three case reports [28,36,37,38]. Abusers mostly were adult males with age ranging from 19 to 67 [28,36,37,38], diagnosed with schizophrenia [28,36,37,38]. The route of administration was always oral, except for one case, which recorded an intramuscular use of benztropine [28]. A maximum drug dose of 120–140 mg has been recorded [36], in association with psychiatric symptoms, including hallucinations, nervousness/agitation, bizarre behavior, confusion and delirium, altered mental state, and flight of ideas [36]. Most important physical symptoms recorded were tachycardia [36,38], hypertension [36,38], urinary symptoms [36,37,38], abdominal pain, and gastrointestinal symptoms [36,37,38]. With regard to the concomitant use of other drugs, the abuse of sedative hypnotics, oral narcotics, heroin, speed, LSD, and alcohol was reported [29]. With regard to the treatment, two cholinesterase inhibitors have been recorded, physostigmine and neostigmine [36,37], and diazepam [28].

### 3.3. Atropine

Three case reports dealt with atropine misuse [39,40,41]. They were represented by two female subjects [39,40] and one male [41], all of adult age. Psychiatric comorbidities recorded were substance use disorder (SUD) [39,40,41] and depression [40]. Regarding the routes of administration, intramuscular [39] and nasal assumption [41] were recorded. Psychiatric symptoms described were agitation, delirium, disorientation [40,41], and anxiety [39]; physical symptoms were rather homogeneous and included tachycardia, tachypnoea, hypertension, dilated pupils, and dry mucous membranes/skin [39,40,41], followed by urinary retention [39], sinus bradycardia, and ataxia [40]. The concomitant use of alcohol [39], cocaine [41], and opioids mixed with atropine was recorded [40]. The use of lorazepam [40,41], activated charcoal [39,41], and naloxone [40] was described for the treatment of atropine abuse.

### 3.4. Scopolamine/Scopolamine N-Butylbromide

Scopolamine was addressed by three studies, a cross-sectional case series with 36 subjects [42], a case series with two subjects [43], and a case report with one subject [44]. All cases were male with age ranging from 15 [43] to 42 years [42]. Only one case had a psychiatric disorder (SUD) [44]. In two studies, the scopolamine was smoked [42,44], while in one, it was taken orally [43]. Psychiatric symptoms included insomnia, irritability, illogical thinking, hallucinations [42], severe agitation, disorientation and aggressive behavior [43], speech problems, and amnesia [42,43]. The physical ones included dry mouth and throat, inhibited bowel movements, palpitation, blurred vision, flushing [42], dry skin, tachycardia [43], and cerebral and lung edema [44]. One article reported on methadone abuse [42] and one on cannabis [44]. Finally, regarding the treatment, one case of scopolamine abuse was treated using midazolam and haloperidol [43].

### 3.5. Biperiden

Two case reports dealt with biperiden misuse in two adult males [45,46]. One of them had no psychiatric comorbidity but had suffered withdrawal syndrome symptoms after discontinuation of the drug [45], while the other suffered from chronic psychosis [46]. The substance was taken orally in one case [45], while in the other intramuscularly at a dosage of 120 mg [46]. One case reported a mild confusional state with spatio-temporal disorientation and psychomotor agitation after the concomitant abuse of THP, cocaine, alcohol, and cannabis was recorded [45]. Elevated hepatic function tests have been reported [46].

### 3.6. Dicyclomine

Dicyclomine was addressed by two case reports [47,48], one involving a 30-year-old male [47] and the other an 18-year-old female [48]. In one case, the misuse was oral at a dose of 50–75 mg/day and concomitant with mefenamic acid [47]; in the other, the misuse was intramuscular [48]. Both studies described withdrawal symptoms with anxiety after drug discontinuation [47,48]; one case also reported depression, anorexia, and confusion [48]. Regarding physical symptoms, in both articles, palpitations, sweating, tachycardia, weakness, blurred vision, and dry skin were recorded [47,48]. Finally, both recorded the treatment done, which consisted of fluoxetine and clonazepam [47] and physostigmine [48].

### 3.7. Orphenadrine

Both studies describing the abuse of orphenadrine were case reports respectively related to a 26-year-old female diagnosed with psychosis [49] and a 24-year-old male with a diagnosis of SUD (amphetamines and cocaine abuse) [50]. In both cases, macrodoses have been reported, up to 1250–1500 mg [50], and symptomatology described included psychotic symptoms with visual hallucinations and mystic–megalomanic delusion, hypomania, agitation, and aggressivity. Physical symptoms included dry and warm skin, mydriasis, asymmetrical abdominal reflexes [49], dizziness, and tremor [50].

### 3.8. Tropicamide

Two articles were related to tropicamide abuse together with other substances, e.g., heroin, benzodiazepines, ecstasy, and cannabis, in three adult subjects [51,52]; interestingly, both described an intravenous route of administration and recorded the following psychiatric symptomatology: relief, euphoria and relaxation [51,52], and hallucinations and dissociation [44,45]. Regarding the treatments adopted, naloxone was administered when tropicamide had been used together with heroin [51]; diazepam and quetiapine were also recorded as long-term treatment [51,52].

### 3.9. Glycopyrronium Tosylate

Only an article reported on the misuse of glycopyrronium tosylate. It was a case report focusing on a 14-year-old female subject diagnosed with ADHD and acne vulgaris who topically took an excessive amount of glycopyrronium tosylate, showing myopia, dry mouth and anhidrosis, urinary hesitancy, and chronic constipation [53].

### 3.10. Oxybutynin

A case series reported on the oxybutynin misuse in two male subjects aged 27 and 45 years, both diagnosed with a SUD, who orally took 100–150 mg/day and 300–400 mg/day of the drug, respectively [54].

### 3.11. Pentolate, Prisoline, and Naphcon-A (Ophthalmic Drug)

An observational study investigated the topical abuse of the ophthalmic formulation including pentolate, prisoline, and naphcon-A in 140 subjects seeking psychotropic effects, including relaxation, pleasure, and increased energy. Side effects were conjunctivitis, eczematoid blepharoconjunctivitis, and conjunctival hyperemia [55].

### 3.12. Procyclidine

A case report dealt with procyclidine abuse in a 36-year-old male subject diagnosed with an antisocial personality disorder, who orally took 40 mg of the drug together with physeptone^®^ (methadone) and alcohol, showing disinhibition, mania, and aggressiveness [56].

### 3.13. Unspecified Anticholinergic Drugs

Finally, a controlled prospective study reported on the abuse of unspecified anticholinergic drugs [57] in 21 subjects (M/F = 14/7) with a mean age of 33.6 ± 6.1, suffering from psychiatric diagnoses, e.g., mood disorder, schizophrenia, schizoaffective disorder, and schizophreniform disorder, and requiring an antipsychotic treatment, who reported effects of relaxation, elevated mood and energy, reduced concentration, visual and auditory hallucinations, confusion, and the physical symptoms such as dehydrated skin, tachycardia, blurred vision, and thirst.

## 4. Discussion

To the best of our knowledge, this work constitutes the first review investigating the diversion and abuse of anticholinergic drugs. These medications block the muscarinic acetylcholine receptor and are usually prescribed for their parasympatholytic effect. Indeed, the effects of inhibition of dopaminergic neurons are normally balanced by the excitatory actions of cholinergic neurons; thus, if dopamine receptors are blocked by antipsychotics, a relative excess of cholinergic activity is caused, resulting in extrapyramidal motor effects, which can be balanced by its block trough anticholinergic agents [58]. On the other hand, anticholinergic drugs also act as a potent indirect dopamine agonist in the limbic system, which can in part explain their misuse potential in both psychiatric and non-psychiatric patients [58,59]. Common anticholinergic agents, such as benztropine, benzhexol/THP, cyclobenzaprine, orphenadrine, and scopolamine, are used for the treatment of both primary and secondary parkinsonism, bradycardia, asthma and chronic obstructive pulmonary disease, dystonia, urinary incontinence, muscle cramps, nausea, and emesis. Moreover, these agents are also frequently seen in the medical setting as instruments of both accidental and intentional overdose [3]. In the present study, they were widely used to treat extrapyramidal motor symptoms caused by antipsychotic drugs or other molecules resulting with antidopaminergic effects [60] and then abused to reach a psychotropic effect, e.g., to abolish neuroleptic-induced anhedonia; conversely, patients might have hypothetically taken more than the recommended dose of anticholinergics in an attempt to treat the adverse effects resulting from the use of antipsychotics [60]. Although muscarinic acetylcholine receptors exist as five subtypes, each with specific characteristics and effects, e.g., M1 subtypes are located on central nervous system (CNS) neurons and sympathetic post-ganglionic cell bodies; M2 receptors are located in the myocardium, smooth muscle organs, and neuronal sites; the M3 muscarinic subtypes receptors are the most common on parasympathetic target tissues, such as in smooth muscle and glandular cells) [59]; finally, the majority of anticholinergic drugs available as medications are non-specific in terms of which receptor subtypes they target, then explaining the rich symptomatology associated with their diversion [61], specifically referring to the psychiatric symptoms resulting from their misuse. In fact, in cases of medication-induced delirium, health care professionals should take into account the possibility of anticholinergic drugs misuse. Indeed, anticholinergic drugs might be abused at clinically and epidemiologically significant levels for their psychotropic effects [3], e.g., to achieve a *high* or euphoria, to elevate energy and mood, to increase social interaction, or to induce an anticholinergic toxic syndrome, which may feature disorientation, hallucinations, paranoia, and confusion [12,24,28]. These clinical symptoms may configure forms of exogenous psychosis, also with chronic developments [62].

Our review confirmed previous literature identifying benzexhol/THP as the most-often abused anticholinergic. This might be related to its greater psychotropic (e.g., stimulatory and euphorigenic) effects [3,4,12,58]. Benztropine and biperiden have also been shown to induce euphoria, owning an abuse potential, albeit less than those of benzexhol/THP [3]. However, benzexhol/THP, benztropine, and biperiden are among the wider available anticholinergics, with differences in the regional diffusion depending on regulatory issues, medicine supply, their promotion and prescription by health care providers, and access to them. These factors may have an influence and increase the base of possible users by encouraging the development of phenomena of abuse.

In most cases, due to its relevant symptomatology, anticholinergic intoxication is often seen and treated in emergency settings. In fact, toxicity symptoms might include dry mouth and mucosal surfaces, mydriasis, decreased bowel sounds, hot and flushed skin, urinary retention, constipation, and agitation, emerge within an hour of ingestion of an acute overdose, and were recorded by almost all studies retrieved. Moreover, tachycardia, hypertension, tachypnoea, and fever are in most cases described, although in severe overdose, hypotension, life-threatening arrhythmias (e.g., supraventricular tachycardias), severe heart blocks, and respiratory depression may occur. Neurological and psychiatric symptoms might include drowsiness, sedation, ataxia, amnesia, and finally coma; and paranoia, hallucinations, delirium, and confusion [1,3]. The diagnosis of anticholinergic intoxication is typically based on the clinical symptomatology presented; moreover, the intravenous use of an acetylcholinesterase inhibitor such as physostigmine can be used as both a diagnostic and a therapeutic intervention [12]. Here, toxicity symptoms are explainable through the pharmacological drug effects related to the antimuscarinic action of the index drug at each target tissue. However, the psychotropic, e.g., euphoric, stimulatory, and antidepressant effects of anticholinergic drugs should still be clarified. From the current findings, both the euphoric and toxic effects are dose-dependent, but it was not possible to understand the eventual threshold dosages related to each drug due to the possibility of personal variations and idiosyncratic reactions related to the use of concomitant drugs and unusual routes of administration [12]. Finally, the chronic use was here related to tolerance and withdrawal phenomena, possibly related to the reinforcing effect of abused drugs on the mesolimbic dopaminergic system, including the ventral tegmental area, the nucleus accumbens, and the prefrontal cortex [58]. Therefore, the rapid discontinuation of an anticholinergic drug was here associated with a withdrawal syndrome characterized by the symptoms including increased anxiety, insomnia, restlessness, sweating, irritability, headache, and tachycardia [16,17,18,25,26,27,30,45]. Moreover, apart from the physical symptomatology, when the drug is withdrawn, abusers might also experience a psychological dependence together with craving, which generally resolve in two weeks [25].

Studies here retrieved have shown that anticholinergic abusers are mostly young, male, single, and, when recorded, unemployed or marginalized, as previously described by the literature [31]. Moreover, anticholinergic drugs are often figured in polydrug abuse since they have been possibly used to potentiate the effects of other psychoactive substances, including alcohol, cocaine, benzodiazepines, and opioids [5,12,17,24,25,28,29,30,31,34,35,40,41,42,44,45,51,52,63]. Indeed, regarding the abuse of anticholinergic medications, three distinct groups of abusers have been previously described [64]: (i) individuals who consume a medication only for its psychotropic and mind-altering effects; (ii) individuals with a medical indication for the use of, e.g., an anticholinergic drug, who might eventually abuse or misuse it for its psychotropic effects; and finally, (iii) individuals who have an appropriate medical indication for the agent and use it according to medical guidelines. Moreover, misusers/abusers might also be recognized because they might exaggerate extrapyramidal symptoms, repeatedly request unnecessary dose increases, or perform doctor shopping practices. In the present review, although in two studies, patients faked extrapyramidal symptoms in order to obtain a prescription for the drug of interest [19,24], sources of the drugs were in all cases licit prescriptions and could then be included in the second group. Accordingly, the European Monitoring Center for Drugs and Drug Addictions (ECMDDA) [2] described the diversion of prescription medicines as one of the new main sources of medicines on the illicit market due to the unsanctioned supply of regulated pharmaceuticals from legal sources, either to the illicit drug market or to a user for whom the drugs were not intended. The EMCDDA also alerted on the increasing online availability of medicines, not only from online licit pharmacies, marketplaces, or suppliers.

Limitations: One of the limitations regarding the literature focusing on prescription drug misuse is both its heterogeneity and the issues in identifying misusing practices. In this regard, considering the United Nations Office on Drugs and Crime (UNODC) definition of misuse of medicines, it could be described as “the problematic consumption outside of acceptable medical practice or medical guidelines, when self-medicating at higher doses and for longer than is advisable, for intoxicating purposes and when risks and adverse consequences outweigh the benefit” [65,66,67]. However, the terminology used in the studies might be variable and inconsistent [3]; thus, in this study, we use misuse as referred to non-medical use, problem use, harmful use, recreational use, self-medication, or inappropriate use, which calls into question whether there is a consensus on the negative consequences (i.e., problem, harm) of their use. Moreover, given the novelty of the topic, the scarcity of articles focusing on the issue should be considered another limitation of the present study. For sure, the heterogeneity of studies recorded, mostly represented by case reports/case series of clinical assessments, interventions, and outcomes, was another important limitation. Moreover, the duration of the studies analyzed and the consequent absence of follow-up evaluations carried out at a distance of time was another limitation of studies retrieved.

## 5. Conclusions

Despite the limitations of the study, the abuse of prescription drugs and medications has rapidly risen, threatening to overtake illicit drugs as the most commonly abused substances. In the present challenging drug scenario, including prescription drugs and medications in general, anticholinergic drugs as substances of abuse should be monitored. Healthcare professionals should be vigilant and prevent possible medicines’ misuse and diversion.

## Figures and Tables

**Table 1:** Main findings of retrieved studies.

Ref (Name, Year)	Country	Study Design	Population	Mean Age (yrs) ± sd	Psychiatric Comorbidity	Medical Comorbidity	Route of Administration and Dosage	Physical Symptoms	Psychiatric Symptoms	Polyabuse	Outcome	Notes
**ATROPINE**												
**Taylor et al., 2007**	North Carolina (USA)	Case report	*N* = 1; F	29	None	None	IM, 4 mg	Tachycardia, tachypnoea, dilatated pupils, dry mucous membranes, dry skin, urinary retention	Anxiety	30 meloxicam tablets, alcohol	Activated charcoal with sorbitol, IV fluids	Symptoms resolved over a 6 h period of observation
**Wang, 2002**	Pennsylvania (USA)	Case report	*N* = 1; F	41	Depression; SUD (heroin addiction)	None	Oral	Pupils dilated, dry skin, sinus bradycardia, ataxia. After IV naloxone: tachycardia, tachypnoea, hypertension	Slurred speech. After IV naloxone: agitation, delirium, disorientation	Codeine and morphine mixed with atropine	IV Naloxone, IV Lorazepam, rapid-sequence intubation, orogastric lavage, and activated charcoal	During hospitalization: persistent agitation refractory to benzodiazepine, pneumonia, and stress gastritis
**Weiner et al., 1998**	Connecticut (USA)	Case report	*N* = 1; M	39	SUD (alcohol and cocaine abuse)	None	Nasal	Tachycardia, hypertension, warm and dry skin, facial flushing, dilated pupils minimally reactive to light, absence of bowel sounds	Agitation, intermittent delirium; disorientation	Cocaine	IV Lorazepam 1 mg, activated charcoal 50 g, cathartic mixture	Patient ingested cocaine adulterated with atropine
**BENZHEXOL/TRIHEXYPHENIDYL (THP)**												
**Crawshaw et al., 1984**	New Zealand	Observational study	*N* = 21; M = 17	21 ± 6	Antisocial personality disorder or socialized conduct disorder (*N* = 8) Schizophrenia (*N* = 7) Borderline personality disorder Depression Bipolar disorder SUD (Alcohol dependence and multiple substance abuse) Adjustment disorder	Extra-pyramidal side-effects of neuroleptic medication in patients with schizophrenia	Oral, 15–60 mg	Dehydration, tachycardia, pronounced thirst, and blurred vision	Toxic-confusional state with psychosis and visual hallucinations, illusions, and distorted time sense (*N* = 10); difficulties in recent memory and new learning were associated with problems of attention and concentration (*N* = 6)	Alcohol, cannabis (*N* = 12), hallucinogens (*N* = 10), opiates (*N* = 10), amphetamines (*N* = 9), benzodiazepines (*N* = 7), cough mixtures, solvents, cocaine, and various neuroleptics	NA	
**Deutsch et al., 1992**	New York (USA)	Case report	*N* = 1; F	33	Adjustment disorder with depressed mood	Cerebral palsy	Oral, maximum 105 mg (21 tablets) in a few hours in each time	Dry skin, dry mouth, and blurred vision	Restlessness, depression, confusion, disorientation, and auditory and visual hallucinations	None	IV lorazepam	Anticholinergic psychosis with hallucinosis
**Fisch et al., 1987**	Israel	Case series	*N* = 14; M = 5	21–30	Borderline personality disorder (*N* = 2); Mixed personality disorder (*N* = 1); Heroin dependence (*N* = 1); Methadone dependence (*N* = 1); Chronic schizophrenia (*N* = 3); Residual schizophrenia (*N* = 6)	None	Oral, 20–60 mg	None	Took THP regularly to achieve a state of hallucinosis; severe paranoid psychosis following the ingestion of 25 mg THP; Sedative and anxiolytic effect; Mildly psychedelic, euphorigenic, and sedative effect; Relieve withdrawal symptoms; euphorigenic effect and hypomanic state; Euphoric and energizing effect; Euphorigenic, stimulating, and socializing effect	Heroin, diazepam	NA	One patient feigned extrapyramidal symptoms in order to obtain THP
**Goggins et al., 1979**	Norway	Case Report	*N* = 1; M	40	Depression Obsessions	Weight loss	Oral 35–40 mg/die	Tachycardia and nausea	Restlessness, euphoria	None	NA	
**Harrison, 1980**	England (UK)	Case Report	*N* = 1; M	19	Anxiety Depression	NA	Oral	Swollen abdomen	Euphoria	NA	NA	
**Kajimura et al., 1993**	Japan	Case report	*N* = 1; M	55	None	Essential tremor	Oral, 20 mg/day for 18 years	Headache, tachycardia, and general fatigue	Memory loss, cognitive impairment. On withdrawal: anxiety, irritability, insomnia, perspiration, and anorexia	None	After stopping THP, clotiazepam and flunitrazepam were used to manage withdrawal	
**Kaminer et al., 1982**	Israel	Case report	*N* = 1; M	30	Chronic paranoid schizophrenia	None	Oral, 30–40 mg/die	Dystonic reaction, withdrawal symptoms, and tachycardia	Acute: auditory hallucinations Chronic: anxiety reduction, sleep disturbance, and euphoria	Cannabis, LSD, amphetamines, barbiturates, and alcohol	Treated with haloperidol 10 mg	The patient used to mix drugs and THP
**Keshavan et al., 1985**	England (UK)	Case report	*N* = 1; M	38	Schizoaffective disorder	None	Oral, 70 mg	NA	Euphorigenic effect, auditory and visual hallucinations	Pimozide 8 mg, lorazepam 2,5 mg day	Admitted to psychiatric ward, gradually reduction of benzhexol with withdrawal symptoms, agitation, depression, and exacerbation of auditory hallucinations	
**Lo et al., 1996**	Taiwan	Case report	*N* = 1; M	35	Chronic schizophrenia	None	Oral, 200 mg/day for 2 years	None	Delusion of reference, thought broadcasting, loosening of association, paralogical thinking, auditory hallucination	None	Decreased dosage of THP and clonazepam	Patient feigned extrapyramidal syndrome to obtain biperiden injection
**Macvicar, 1977**	California (USA)	Case report	*N* = 1; M	30	Paranoid schizophrenia	None	Oral 24–30 mg/die	Dry mouth and constipation	Toxic psychosis, hallucinations, euphoria, and talkativeness	NA	NA	
**Mahal et al., 2018**	Delhi (India)	Case series	*N* = 2; M	32	Delusional Disorder	1st case: headache, recurrent abdominal pain; 2nd case: none	Oral, 40–60 mg	1st case: headache, sweating, and tachycardia; 2nd case: none	Drug abuse aimed to obtain relaxation; withdrawal symptoms were restlessness, irritability, and aggressiveness	1st case: nicotine and alprazolam; 2nd case: nicotine and opioid	NA	History of multiple prescription drug misuse
**Mclnnis et al., 1984**	Iceland	Case series	*N* = 2; M = 1	25 and 35	Drug and alcohol abuse Schizophrenia	None	Oral, 40 mg	Ataxia, headache, visual difficulties, and photophobia	Confusion, self-harm, disorientation, agitation, bizarre behavior with difficulties of speech, and reduced concentration	Haloperidol, benzodiazepines, alcohol; perfenazine (100 mg IM every 2 weeks), chlorpromazine 50 mg, and clomipramine	NA	
**Michael et al., 1984**	Karnataka (India)	Case series	*N* = 2; M	28 and 30	Schizophrenia	None	Oral, 6 mg	None	None	Trifluoperazine 15–20 mg, chlorpromazine 300–600 mg	Withdrawal symptoms including agitation, tachycardia, restlessness, aggressiveness, lethargy, giddiness, sweating, and craving	
**Mohan et al., 1981**	Delhi (India)	Case report	*N* = 1; M	35	Chronic depression	None	Oral 120–140 mg/die	NA	Visual hallucinations, paranoid ideas, and ideas of reference	NA	NA	
**Nappo et al., 2005**	São Paulo (Brazil)	Survey	*N* = 37; M = 29	20–30 yy =14; 30–40 yy =18; >40 yy = 5	None	NA	Oral, from one-half to four pills (3–8 mg)	Dry mouth, gastritis, vomiting, tachycardia, urinary retention, and dental caries	Euphoria, hallucinations, delirium, nervousness, aggressiveness, memory loss, decreased attention, loss of appetite, insomnia, and depression	Alcohol, coffee, nicotine	NA	THP was neither the initial drug in the substance user’s career nor their main drug
**Petkovic et al., 2012**	Serbia	Case report	*N* = 1; F	59	Persistent delusional disorder	None	Oral, 15 mg	NA	NA	None	Death	THP blood and urine concentrations were those associated with fatalities
**Qureshi, 1992**	Saudi Arabia	Observational study	*N* = 14; M = 13	27.93 ± 6.55	Schizophrenia (*N* = 9) Schizoaffective disorder (*N* = 1) Mood disorder (*N* = 3) Factitious disorder (*N* = 1)	NA	Oral	Tardive dyskinesia, extrapyramidal symptoms	Symptoms of withdrawal included palpitations, restlessness, body aches, lethargy, irritability, aggression, discomfort, craving, and anxiety	Polydrug abuse (57.14%)	THP on a prophylactic basis with improvement in negative symptoms	
**Qureshi et al., 1997**	Saudi Arabia	Case control	*N* = 30; M = 25	33.83 ± 7.4	Drug abuse (*N* = 23) Unspecified mental disease (*N* = 18) Schizoid (*N* = 16)	NA	Oral	Dyskinetic movements	Drug abusers were characterized by less negative symptoms	Some 53% abused beverages, amphetamines, and benzodiazepines	NA	
**Rao et al., 2014**	Karnataka (India)	Case report	*N* = 1; F	55	Psychotic disorder	None	Oral	NA	Increased speech output, psychomotor agitation, and reduced need for sleep	None	Treated with clonazepam, haloperidol 10 mg/day, and THP 4 mg/day	
**Rubinstein, 1979**	California (USA)	Case series	*N* = 8 (*N* = 6 related to THP), 4 M, 2 F	25–32	Schizoaffective disorder Schizophrenia	None	Oral, 15–250 mg/die	Eye-rolling and finger stiffness	When recorded (cases 2–3), bizarre and violent behavior, difficulty in speech, and hallucinations were described; in some cases, high-dosage THP was taken to get high	Diazepam, LSD, amphetamines, thiothixene, cannabis, and PCP	Case 5 was treated with diazepam and fluphenazine for PCP-induced toxic psychosis	Most of the patients continued to ask for THP during the hospitalization
**Sofair et al., 1983**	New York (USA)	Case report	*N* = 1; M	24	Chronic depression	None	Oral, 60 mg/die	Dry mouth, constipation	Relaxation and impaired concentration	THP addiction	Abrupt cessation of THP	
**Thunyapipat et al., 2018**	Thailand	Case series	*N* = 27, M = 15	14.2 (range 10.6–21)	Mental health comorbidity ADHD Conduct disorder Learning and intellectual disability	NA	Oral, from 1 to 50 tablets once	No peripheral anticholinergic symptoms	Agitation, hallucination	Unspecified drug abuse (63.6%)	Motivational interviewing in 68.4% of hospitalized cases	Of those who received motivational interviewing, all discontinued abusing THP at a monthly follow-up visit
**Torrents et al., 2018**	France	Cohort study	*N* = 69; M = 67, F = 2	36	Unspecified psychiatric disorder (*N* = 4)	NA	Oral	NA	The abuse aimed to reach anxiolytic, sedative, and stimulating effects or to control the use of another drug; reported side effects were behavioral disorders such as aggressiveness, agitation, and paranoia	Tobacco (72%), benzodiazepines, cannabis, alcohol	Not reported	
**Younis et al., 2009**	United Arab Emirates	Observational study	*N* = 190	29.5 (19–52)	Intermittent explosive disorder OCD	NA	Oral	NA	The abuse aimed to relax and control aggressive outbursts	Alcohol, cannabis, cocaine, and opioids	95 patients had difficulty stopping taking benzhexol	
**Zemishlany et al., 1996**	Israel	Case control	*N* = 14; M = 11	34 ± 5.3	Antisocial personality disorder (*N* = 3)	NA	Oral, >20 mg	NA	Thinking disturbance, withdrawal retardation, hostile suspiciousness, anxious depression	Cannabis and alcohol	Neuroleptic treatment	
**BENZTROPINE**												
**Craig et al., 1981**	Colorado (USA)	Case series	*N* = 2; M = 1	19 and 22	NA	None	Oral, 120–140 mg	1st case: dilated pupils, dry skin, urinary retention; 2nd case: tachycardia, fever, hypertension, dilatated pupils, warm and dry skin	1st case: agitation, bizarre behavior, altered mental status; 2nd case: hallucinations, flight of ideas, agitation, combativeness	1st case: abuse of sedative-hypnotics and oral narcotics; 2nd case: speed, LSD, alcohol	1^st^ case: treated with physostigmine, discharged 18 h later; 2nd case: treated with physostigmine	Diagnostic trial of physostigmine
**Esang et al., 2021**	Pennsylvania (USA)	Case report	*N* = 1; M	67	Schizophrenia	Benign prostatic hyperplasia; essential hypertension	Oral	Hematochezia, abdominal pain, constipation, and difficulty with urination	None	None	Spontaneous remission of symptoms after returning to therapeutic doses	
**Isbister et al., 2003**	Australia	Case report	*N* = 1; M	33	Schizophrenia Borderline personality disorder Recorded recreational use of benztropine	Hypertension; epilepsy; cluster headache	Oral, 27 tablets (2 mg each) during the previous 6 days	Abdominal pain, distention, drowsiness, hypertension, tachycardia, blurred vision, anticholinergic-induced ileus with absent bowel sound	Confusion, hallucinations, delirium	NA	IV fluids, meperidine, IV neostigmine 2 mg + 2.5 mg	
**Rubinstein, 1979**	California (USA)	Case report	*N* = 8 (*N* = 2 were related to benztropine, M)	26 and 28	Schizophrenia	NA	Oral/IM	Stiffness and eye rolling	Nervousness	Drug abuse (heroin, alcohol, unspecified)	Treated with thioridazine and diazepam	
**BIPERIDEN**												
**Affaticati et al., 2015**	Italy	Case report	*N* = 1; M	27	None	Withdrawal syndrome symptoms, e.g., headache	Oral 16 mg/die	Urinary retention	Mild confusional state with temporal, spatial disorientation, impairment of attention and concentration, psychomotor agitation	Trihexyphenidyl, cocaine, alcohol, cannabis	Biperiden gradually tapered; the patient was also treated with quetiapine, 50 mg/d	After 6 months, he stopped using biperiden
**Ozucelik et al., 2007**	Turkey	Case report	*N* = 1; M	52	Chronic psychosis	None	120 mg IM (60 biperiden tablets)	Swelling and pain caused by an abscess in injection site. Mildly elevated hepatic function tests	NA	NA	General surgery for abscess drainage and antibiotics	
**DICYCLOMINE**												
**Sinha et al., 2020**	Chandigarh (India)	Case report	*N* = 1; M	30	None	Dysmenorrhea, headache	Oral 10–15 tablets per day, 50–75 mg/day	Tachycardia, palpitation, sweating	Withdrawal symptoms, anxiety	Dicyclomine and mefenamic acid	Reversion to normal physiological state in a week after being treated with fluoxetine 20 mg per day and clonazepam 0.5 mg per day	
**Das et al., 2013**	Bengal (India)	Case report	*N* = 1; F	18	None	None	IM	Weakness, palpitation, fever, blurred vision, sweating, dry skin	Confusion, withdrawal reaction, anxiety, depression, anorexia	None	Treated with IV physostigmine with remission in a week	Drug use began with drug treatment for enterocolitis
**GLYCOPYRRONIUM TOSYLATE**												
**Tarr et al., 2021**	Bronx (USA)	Case report	*N* = 1; F	14	ADHD	Acne vulgaris	Topical	Progressive myopia, dry mouth, anhidrosis, urinary hesitancy, and chronic constipation	None	None	Discharged at home after monitoring spontaneous remission	Patient would possibly have access to other medication when unsupervised
**ORPHENADRINE**												
**Nissen et al., 1987**	Norway	Case report	*N* = 1; F	26	Psychosis (alcohol-induced psychotic reactions)	None	Oral, 800 mg	Dry and warm, pupils dilated, asymmetrical abdominal reflexes	Disorientation, clouding of consciousness, agitation, aggressivity, pressured speech and laughing with loosening of associations, psychosis with mystic-megalomanic delusion and visual hallucinations	Levodopa 2000 mg; benserazide chloride 400 mg; alcohol	Admitted to psychiatric ward	
**Schifano et al., 1988**	Italy	Case report	*N* = 1; M	24	SUD (amphetamines and cocaine abuse)	Not reported	Oral, up to 1250–1500 mg/day over a period of 2 months	Dizziness, tremor	Euphoria, visual hallucinations, mood enhancement, unpleasant misperceptions	NA	Drug stopped because difficult to obtain	Haloperidol and orphenadrine prescribed for hallucinations
**OXYBUTYNIN**												
**Balasar et al., 2016**	Turkey	Case series	*N* = 2; M	27 and 45	SUD (alcohol and substance abuse)	overactive bladder	Oral, 100–150 mg/day and 300–400 mg/day	Xerostomia, constipation, urinary arrest	Relief from the need of using drugs and alcohol	None	NA	
**PENTOLATE, PRISOLINE, AND NAPHCON-A (OPHTHALMIC DRUG)**												
**Al-Khalaileha et al., 2019**	Jordan	Observational study	*N* = 140; M:79; F: 51	<20 yy: 13; 21–40 yy: 81; 41–50 yy: 29; 50–60 yy: 5; >60 yy: 2	NA	NA	Topical	Conjunctivitis, eczematoid blepharoconjunctivitis, and conjunctival hyperemia	The abuse was aimed to relax, get high, induce pleasure, and boost energy	NA	NA	*N* = 19 cases have been suspected for abuse
**PROCYCLIDINE**												
**Coid, 1982**	England (UK)	Case report	*N* = 1; M	36	Antisocial personality disorder	None	Oral 40 mg	NA	Disinhibition, mania, aggressiveness	Physeptone^®^ (methadone), alcohol		
**SCOPOLAMINE/SCOPOLAMINE N-BUTYLBROMIDE**												
**Jalali et al., 2014**	Iran	Cross-sectional case series	*N* = 36; M	27–42	None	None	Smoked tablets	Dry mouth, dry throat, bowel movement, palpitation, blurred vision, flushing	Insomnia, irritability, inability to concentrate, incoherent speech, slurred speech, amnesia, illogical thinking and hallucinations	Substance abuse (on methadone)	Not reported	
**Kummer et al., 2015**	Germany	Case series	*N* = 2; M	16 and 15	None	None	Oral 40 mg una tantum	Flushed and dry skin, tachycardia	1st case: severe agitation, disorientation, intermittent aggressive behavior, nonsensical speech; 2nd: partial amnesia	None	Transferred to intensive care unit, he was treated with midazolam and haloperidol; both were discharged from hospital 2 days later	
**Strano-Rossi et al., 2021**	Italy	Case report	*N* = 1; M	41	SUD	Multiple traumas from aggression	Smoked tablets	Cerebral and lung edema	NA	Cannabis	Death	Other drugs identified in urine and blood included benzodiazepines, antipsychotic drugs in therapeutic or subtherapeutic concentrations
**TROPICAMIDE**												
**Bozkurt et al., 2014**	Turkey	Case series	*N* = 2; 1 M; 1 F	37–38	None	None	IV	1st case: decreased appetite, weight loss, and blurred vision; 2nd case: palpitations and sweating	1st case: the patient experienced relief and relaxation mixing tropicamide and heroin; hallucinations and dissociation were recorded; 2nd case: dissociation, anxiety, and concentration problems	1st case: cocaine, clonazepam, ecstasy, cannabis, and heroin; 2nd case: alprazolam, codeine, cocaine, and ecstasy	1st case: treated with naloxone and opioid withdrawal symptoms; discharged after 14 days of hospitalization; 2nd case: discharged with quetiapine 100 mg/day treatment	Tropicamide was mixed with heroin
**Spagnolo et al., 2013**	Italy	Case report	*N* = 1; F	22	None	None	IV	Palpitations, hypertension, tachycardia, fever, mydriasis, warm and flushed skin, and xerostomia	Euphoria, hallucinations	Heroin	Treated with diazepam	
**UNSPECIFIED ANTICHOLINERGIC DRUGS**												
**Wells. et al., 1990**	Tennessee USA)	Controlled prospective study	*N* = 21; M = 14	33.6 ± 6.1	Mood disorder (*N* = 3) Schizophrenia (*N* = 15) Schizoaffective disorder (*N* = 2) Schizophreniform disorder (*N* = 1)	NA	NA	Dehydrated skin, tachycardia, blurred vision, and thirst	Relaxation (*N* = 17), elevated mood (*N* = 15), energy (*N* = 14), reduced concentration (*N* = 11), visual hallucinations (*N* = 4), confusion (*N* = 3), and auditory hallucinations (*N* = 3)	Antipsychotics	NA	

Abbreviation: ADHD—attention deficit hyperactivity disorder; F—female; M—male; DPT—drug provocation tests; HC—healthy control; IM—intramuscular; IV—intravenous; LSD—lysergic acid diethylamide; N/A—not applicable; PCP—phencyclidine; SD—standard deviation; SUD—substance use disorder.

## Data Availability

Not applicable.
